# Multidomain and multilevel strategies to improve equity in maternal and newborn health services in Nepal: perspectives of health managers and policymakers

**DOI:** 10.1186/s12939-023-01905-7

**Published:** 2023-05-26

**Authors:** Resham B Khatri, Yibeltal Assefa, Jo Durham

**Affiliations:** 1 School of Public Health, Faculty of Medicine, the University of Queensland, Brisbane, Australia; 2 Health Social Science and Development Research Institute, Kathmandu, Nepal; 3 School of Public Health and Social Work, Queensland University of Technology, Brisbane, Australia

**Keywords:** Underlying challenges, Multidomain, Multi-level, Inequity, Maternal and newborn health services, Nepal

## Abstract

**Background:**

Nepal has committed to achieving universal coverage of quality maternal and newborn health (MNH) services by 2030. Achieving this, however, requires urgently addressing the widening inequity gradient in MNH care utilisation. This qualitative study examined the multidomain systemic and organisational challenges, operating in multi-level health systems, that influence equitable access to MNH services in Nepal.

**Methods:**

Twenty-eight in-depth interviews were conducted with health policymakers and program managers to understand supply-side perspectives of drivers of inequity in MNH services. Braun and Clarke’s thematic approach was employed in analysing the data. Themes were generated and explained using a multidomain (structural, intermediary, and health system) and multi-level (micro, meso and macro) analytical framework.

**Results:**

Participants identified underlying factors that intersect at the micro, meso and macro levels of the health system to create inequity in MNH services. Key challenges identified at the macro (federal) level included corruption and poor accountability, weak digital governance and institutionalisation of policies, politicisation of the health workforce, poor regulation of private MNH services, weak health management, and lack of integration of health in all policies. At the meso (provincial) level, identified factors included weak decentralisation, inadequate evidence-based planning, lack of contextualizing health services for the population, and non-health sector policies. Challenges at the micro (local) level were poor quality health care, inadequate empowerment in household decision making and lack of community participation. Structural drivers operated mostly at macro-level political factors; intermediary challenges were within the non-health sector but influenced supply and demand sides of health systems.

**Conclusions:**

Multidomain systemic and organisational challenges, operating in multi-level health systems, influence the provision of equitable health services in Nepal. Policy reforms and institutional arrangements that align with the country’s federalised health system are needed to narrow the gap. Such reform efforts should include policy and strategic reforms at the federal level, contextualisation of macro-policies at the provincial level, and context-specific health service delivery at the local level. Macro-level policies should be guided by political commitment and strong accountability, including a policy framework for regulating private health services. The decentralisation of power, resources, and institutions at the provincial level is essential for technical support to the local health systems. Integrating health in all policies and implementation is critical in addressing contextual social determinants of health.

**Supplementary Information:**

The online version contains supplementary material available at 10.1186/s12939-023-01905-7.

## Introduction

From Ayurveda medicine to one that incorporates western-based health care system, Nepal has continuously implemented reform with the intent of improving coverage of quality care. Reforms have been driven by internal factors, including political reforms, economic development, migrant labour movements, and changing disease patterns, including an increasing non-communicable disease (NCD) burden and changes in demand. Health system reform has also been driven by global norms and targets, including the Alma-Ata and Astana declarations, and the Millennium and Sustainable Development Goals (SDGs) [1–3]. Current policies include financial subsidies for citizens defined as poor, to treat some NCDs, a safe motherhood programme covering free institutional delivery and incentives for mothers and health workers, free antenatal and postnatal care at peripheral facilities and district hospitals, community-based integrated management of neonatal and childhood illnesses, and a gender equity and social inclusion strategy in health [4]. A National Health Insurance Program (NHIP) is being implemented to progress universal health coverage (UHC) [5, 6].The 2015 constitutional shift that transformed Nepal from a unitary governance system to a federal republic with seven provinces, sub-divided into local governments (municipalities) contributed to further reform to align health systems with the new federal structure [7].

The various reforms, alongside socioeconomic uplift, have contributed to significant improvements in maternal and newborn health (MNH) outcomes, despite an armed conflict between 1996 and 2006. For instance, from 1996 to 2022, Nepal’s Neonatal Mortality Rate (NMR) reduced from 50 (per 1000 live births) to 21 [8], and its Maternal Mortality Ratio (MMR) reduced from 539 (per 100,000 live births) to 281 from 1996 to 2016 [9]. Progress, however, has not been even. Neonatal deaths remain high, and disparities based on caste, socioeconomic position and geographic location are evident. For example, the 2016 Nepal Demographic Health Survey (NDHS) reported that only 66% of mothers from the poorest wealth quintile accessed institutional delivery compared to 98% of mothers from the wealthiest wealth quintile [8]. Similarly, our analysis of NDHS 2016 revealed coverage of at least four antenatal care (4ANC) visits, institutional delivery, and at least one postnatal care (PNC) visit within 24 h of childbirth among women with triple forms of disadvantages (poor and illiterate and ethnic minority) was 48%, 40%, and 27% respectively compared to 93%, 93%, and 78% among women with triple forms of advantage [10]. Commonly cited reasons for these disparities are geographical difficulties (e.g., terrain, distance, road conditions, access to transport), lack of human resources and the direct and indirect cost of attending health services [11–13].

Since the shift to a federal system, reducing inequities in health have been hampered by a lack of clarity on decentralisation, continued centralisation of resources, and poor coordination between federal and provincial authorities, exacerbating pre-existing challenges, such as inadequate and maldistribution of human resources, poor supply of essential medicines and equipment, and limited accountability [14]. The federal government, for example, retains control of 60% of the health budget, which is mostly spent on procurement [14, 15] and distribution of healthcare workers, including transfer, promotion, and deputation, remains centralised, limiting opportunities for tailoring services to population needs. Furthermore, while the shift from public, to a mix of private and public health services, has improved access, this has predominantly benefited people living in urban areas and of middle and higher income position [16], with high out-of-pocket (OOP) expenses further contributing to inequities in access for poorer households [17]. People must pay from OOP expenditure for these services in private facilities where they prefer visits and pay in favour of perceived better quality of care[18, 19]. Aside from private pharmacies, public facilities are most commonly used in rural areas.

Equity in health is important in enabling everyone to reach their full potential and relates to social justice. Health equity is defined as an absence of unfair and avoidable or remediable differences in health among populations or groups defined socially, economically, demographically or geographically [20–23]. It is concerned with the allocation of resources and power and recognises the obligations of those who have power over the distribution of resources, rights, and opportunities to share these resources proportionately and fairly [24–27]. To achieve health equity, it is imperative to understand the factors that influence the health system’s organisation, policy and governance and beyond to create health (in)equities.

To understand drivers of health inequities in Nepal, research has predominantly explored user-level barriers to service utilisation, availability and quality services. While important, as service delivery and use at the local level is influenced by higher-level organisational and systemic factors [28], it is also necessary to understand how macro-level policy and governance interacts with the micro, meso and macro level to create health (in)equities. To address this, the current study qualitatively examined from managers and policymakers’ perspectives the multidomain systemic and organisational challenges, operating in multi-level health systems, that influence equitable access to MNH services in Nepal. Before proceeding however, to contextualise the paper, an overview of the current health system in Nepal is provided.

### Socioeconomic and health care system context of Nepal

Nepal is ethnolinguistically diverse (29 million population) with 125 ethnic groups and 123 languages [4]. Nearly one-third (29%) of total populations are below the multidimensional poverty index [29] with disparities seen based on geographic location and caste. While legally abolished, a socially constructed multilayered caste system continues to exist in practice. Within this caste system, compared to Brahmin and Chettri (considered as upper caste groups, and dominant Nepali-speaking populations), Dalits (often referred as untouchable caste), Janajati (indigenous), and Madhesi (resident living Terai region) ethnic groups have lower socioeconomic status and poorer access to health services. Dalits comprising nearly 13% of the population are the most socially disadvantaged caste groups [30]. People from the Madhesi ethnic community, the primary population of Madhesh province, which has one of the highest scores on the multidimensional poverty [31], experience socioeconomic marginalisation and poor access to healthcare with most services provided in Nepali, rather than Maithali and Bhojpuri, the commonly used languages of ethnic Madhesis [4]. Additionally, indigenous populations such as Tamang in the Hill region, and Tharu in the Terai region are marginalized with limited access to health services[31]. People living in remote municipalities of all provinces also face geographical barriers in accessing health facilities, including MNH services due to limited transport and irregular availability of services [11].

Nepal’s health services are a mix of public and private services under a decentralised health system: the first tier, including a network of Female Community Health Volunteers (often referred to as PHC services), is governed by local governments [7, 32],the second tier (services include secondary care) is governed by the provincial level, and the third tier (specialised care) health system is governed by the Federal Ministry of Health (Federal MOH) [31]. Private facilities mainly provide tertiary-level health services in urban areas and are paid for through OOP payments or private insurance. The federal MoHP uses a top-down approach of policy and planning [33], with most of the health budget retained at the federal level [5]. Since 2017, health staff have been able to choose whether to work at the federal or lower-levels governments, with most staff skilled in program management remaining at the federal level [34].

Each municipal health section is staffed with a health coordinator and assigned to manage public health programs. Despite the shift to a federal system, however, there has been no fundamental policy and institutional reform per constitutional mandates in the provincial and federal health systems [35]. For example, the previous regional health directorate was renamed the Provincial Health Directorate (PHD) and is under the de-facto jurisdiction of the federal health system. In addition, a health division was established under the provincial Ministry of Social Development (MoSD)[36]. The PHD is based on the provincial level but is under the federal MoH (implicitly). Thus, the MoSD’s health section and PHD (federal MoH) are facing a potential clash in chain command [52]. At the federal level, there are several divisions (e.g., Policy and Planning Division) for policy issues and the Department of Health Services and its divisions (e.g., Family Welfare Division) for the implementation/operational wings of the MoHP [36].

## Methods

### Study design and study participants

We conducted in-depth interviews with MNH policymakers and managers (Supplementary file, Tables S1 and S2) to explore their perspectives on the underlying challenges of inequity in health services. Participants were selected based on their experience and ability to provide insights into multi-level health systems challenges that create inequity in health services [26]. Participants included key policy managers, non-governmental organisations (NGOs) professionals, and civil society members engaging in private providers. To be invited to participate, each potential participant had to have had at least two years’ experience. Participants were also selected based on pragmatic considerations (e.g., availability during data collection, the first author’s (RBK) own experience and personal networking with them). In total 28 participants were recruited, with fifteen coming from the federal level, six from the provincial level, and seven from local governments (Table 1). Sample size selection was guided by the principle of information saturation [37], and based on informational redundancy or saturation [38]. The diversity of participants’ experiences allowed for a range of perspectives on different levels of the health system.

**Table 1:** Description of study participants for in-depth interviews

Level/sector	Public sector	Non-government sector
Federal level (15)	Child health section chief, Ex-director General, Safe Motherhood Section Chief, Member (Nepal Nursing Association), Professor (Pediatrics) (7).	INGOs (One Heart Worldwide, Save the Children, Care Nepal, Plan Nepal, Hellen Killer International), MNH focal person (United Nation Children’s Fund, WHO, DFID, USAID’s SSBH Program) (8).
Provincial level (6)	Chief of health (MOSD), Director (Public Health Directorate), PHO (Jumla) (3)./	INGOs: Save the Children, Plan Nepal (3).
Local level (7)	(District PHO, local health officer) (6).	INGO: Plan Nepal (1).

PHO: Public Health Officers. INGO: International Non-Governmental Organisation

### Data collection

An interview guide (Supplementary file, Table S3) was developed and pre-tested with three health professionals working in Nepal (pre-tested samples were excluded from the analysis). Some modifications were made, particularly regarding language style, the flow of questions, and possible additional probing questions. In-depth interviews were conducted in Nepali from November 2018 to January 2019. Interviews were digitally recorded and subsequently transcribed into English.

### Data analysis

We used Braun and Clarke’s six-step thematic analysis approach [39–41]: (1) familiarising with data; (2) generating initial codes; (3) searching for themes; (4) reviewing themes; (5) defining and naming themes; and (6) producing the report. The first author (as a bilingual and native Nepali speaker) conducted, and audio-recorded all in-depth interviews. A professional transcriber transcribed all digitally recorded interviews in Nepali, and another professional translator later translated later transcripts into English. To familiarise with data, the first author listened to audio recordings, followed by a reading of the hard copy of the Nepali transcriptions to check whether the transcriptions captured the audio records. Important ideas were marked up in each print copy transcript, and codes were developed in the annotated printed copy transcription. Codes were marked up in the hard copy of the Nepali transcripts were identified in English transcripts of each interview in the digital copy in NVivo. All codes and data extracts were read, with similar codes grouped into subthemes. Subthemes with similar ideas were grouped again to generate themes. Finally, the generated themes were mapped in the multidomain-multilevel framework. All electronic copies of the translated interview files were imported into NVivo software Version 12 (QSR International Pty Ltd).

### Conceptual framework

**Fig. 1:**
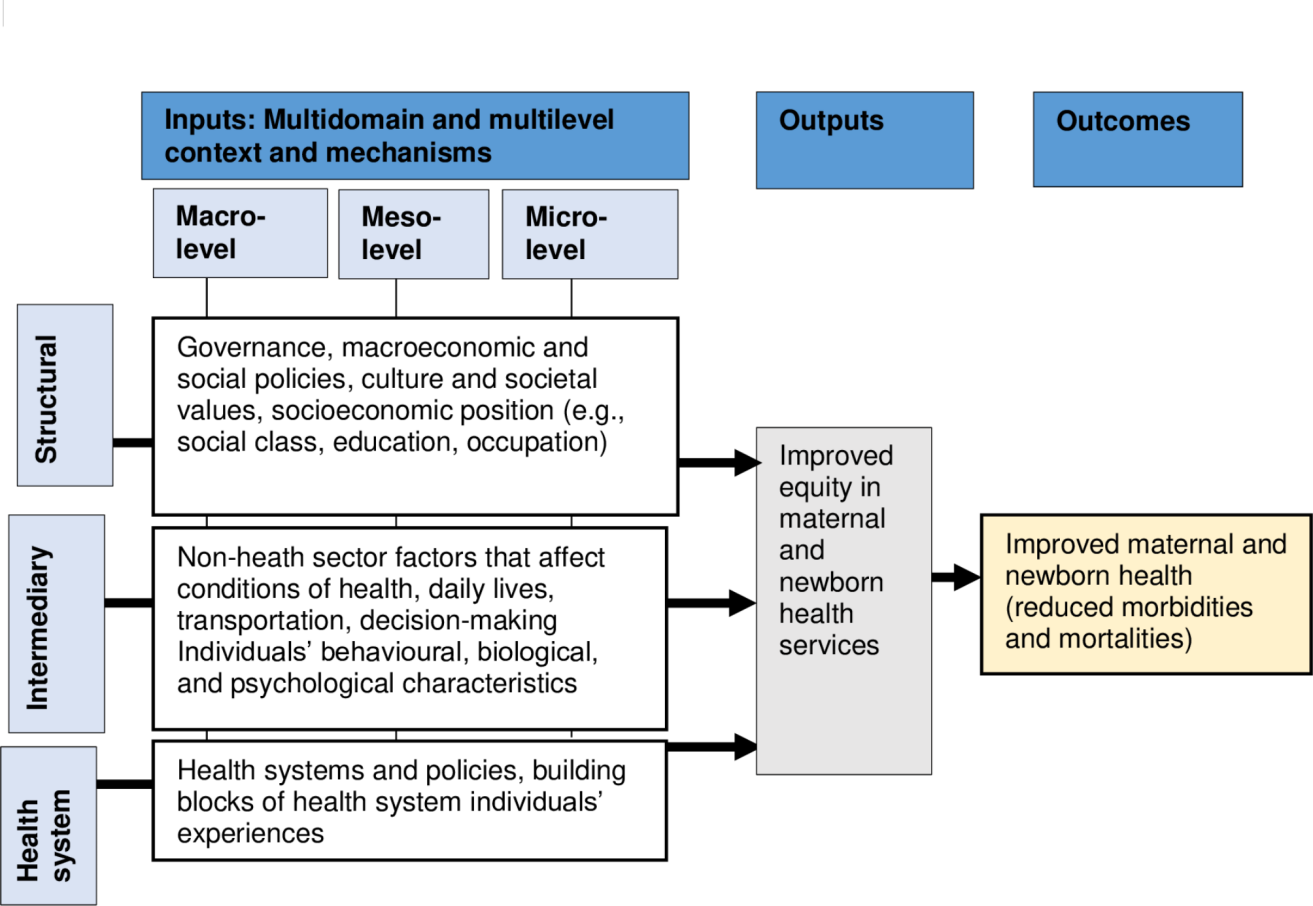
A conceptual framework to guide the data analysis

We derived the concept of inputs (contexts and mechanism)-outputs-outcomes model [Fig. 1] adapted from the previous frameworks [42, 43].

Inputs cover mechanisms and contexts of several underlying challenges under structural, intermediary and health system domains. The structural domain covers all distal challenges, i.e., all basic sociopolitical challenges such as governance, social policies, and socioeconomic positions. The intermediary domain covers intermediate (non-health sector) challenges that usually influence health conditions through living and working conditions, changing individuals’ behaviour and care-seeking practices. Finally, the health system or proximal challenges include factors affecting the production and supply of health services and influence intermediate and proximal challenges in achieving equity in access to MNH services.

The underlying multidomain challenges can operate at the macro, meso and micro-levels [42] and intersect at multiple levels, although the intensity of influence may vary at different levels. The macro-level is the higher-level system (which involves stakeholders and institutions at the federal level) that designs policies and allocates resources [43]. At the meso-level, provincial stakeholders and institutions are involved in the contextualisation of macro-policies into programs and strategies [44]. Finally, at the micro or local level, the system of institutions and stakeholders where programs and strategies are delivered in services and interventions [31] and where health workers and users’ interface with each other [45]. Together, the input interactions of all domain-specific contexts and mechanisms produce the output of access to and use of health services and the extent to which these outputs are equitable.

### Research ethics

Ethical approval was obtained from the Human Ethics Committee of the University of Queensland (UQ) and the human research ethics committee of the Nepal Health Research Council. Before conducting in-depth interviews, informed written consent was taken from each participant. In-depth Interviews were provided with monetary incentives such as transportation costs for compensation for partcipants’ time. Such incentive was provided to increase study participants’ commitment to being interviewed for the study.

### Findings

Based on the subjective views of study participants, our analysis generated twelve underlying factors that drive inequity in MNH services. Of the twelve, six were structural, and three of each were intermediary and health system challenges. These challenges were collated at the macro, meso, and micro-levels, with their relative influence at various levels indicated by symbol “+”.

**Table 2:** Multidomain-multilevel challenges in equity in MNH services in Nepal, 2019

Themes (domain)	Marco-level	Meso-level	Micro-level
Provision of poor-quality MNH services (HS)	+	+	+++
Poor empowerment in decision making on health services (I)	+	+	+++
Inadequate community participation and engagement (S)	+	+	+++
Limited contextualisation of non-health sector programs (I)	+	+++	++
Inadequate evidence-informed health programs (HS)	+	+++	+
Weak decentralisation in the health sector (S)	++	+++	++
Weak health planning and management (HS)	+++	++	+
Inadequate collaboration for health in all policies (I)	+++	++	++
Politicisation of health workforces (S)	+++	++	+
Unregulated private health services (S)	+++	++	+
Weak digital governance and institutionalisation of policies (S)	+++	++	+
Corruption and limited accountability in health sector (S)	+++	++	+

S = structural; I = intermediary; HS = health system challenges. “+” = 25–30% of the total study participants agreed. Themes 1-3, 4-6, 7-12 indicate perceived high influence at the micro, meso and macro levels, respectively

Table 2 shows several challenges within the health systems perceived to be contributing to inequity in health services. At the local level (micro-level), challenges identified were poor quality health care, access barriers including cultural and financial constraints, poor community participation, and poor empowerment and engagement. Local-level challenges were reported to affect health service users and health care providers through formal health care institutions and informal value systems (sociocultural values and norms). Challenges at the local level were reported to be due to poor performance of provincial health systems, especially a lack of contextual operationalisation of policies. Three main challenges that influence the operationalisation of federal health policies at the provincial level system. While organisational weaknesses at the provincial level influence the local health systems.

The macro (federal) challenges included poor management and governance, policy, and systemic issues. The current analysis identified macro-level challenges related to higher-level management, policy and structural factors concerned with power and resources. These factors affect health systems and organisations, primarily at the federal level, and can influence at the lower level. For instance, the politicisation of health workforce can influence the provincial and local health systems. At the local level, major challenges were the poor provision of quality care, poor access to health services, and inadequate community communication in local health systems. These multi-level intersecting challenges operate at different levels, as explained in the following subsections.

### Local level challenges

At the local level, there were three key challenges (poor quality of care, lack of empowerment, and inadequate community participation). However, those challenges intersect at a higher level.

#### Provision of poor-quality MNH services (HS)

At the micro-level, most participants explained quality of care was sometimes compromised with women, for example, being discharged earlier less 12 h after institutional delivery, due to a lack of beds and facilities in the health posts. In rural areas, health facilities were reported to have inadequate infrastructure, experience commodity stock-outs, and unable to provide continuous service or a functional referral system. One local officer said:

*Poor infrastructure of health facilities is a problem for quality services as many rural facilities lack basic requirements (e.g., rooms, beds, ambulance services, medicines and supplies, and accommodation). So, pregnant women do not prefer to visit those facilities while referral hospitals have overcrowded. (L_5_GO)*

Another participant explained how the inability to provide continuous/uninterrupted service, could also lead to a loss of trust in the services:

*If a woman visits and finds a health facility was closed, she must return home and decide not to visit next time. If such happens frequently, the communities’ trust will go in vain. (L_5_GO)*

Most participants felt that health workers’ program management skills (data analysis and use in health planning) competency levels also contributed to poor-quality MNH services.

*Health coordinators lack evidence-based planning and management skills; they have low academic qualifications and data analysis skills. (Fed_11_GO)*

Other reasons provided to poor quality of care related to inefficient coordination and referral mechanisms, limited community based follow up once women were discharged, and a focus on technical aspects of care rather than their care experience. Together these factors, alongside the social determinants of health, were said to contribute to women dropping out along the continuum of maternity care, especially in rural areas.

*Community health programs have not been appropriately implemented. Without linking hospitals and community programs, maternal deaths cannot be prevented. When women return home from facilities after childbirth, follow-up visits should be done to improve social determinants (e.g., housing, water, hygiene, and sanitation). (Fed_4_UN)*

A few participants said health providers do not always adhere to quality care standards. One participant explained what happens after C-section delivery:

*After C-section delivery, obstetrician said to nurse, ‘Okay, keep that baby and show/handover to the visitor/caretaker’. Ideally, nurse-midwives can conduct normal deliveries but lack the necessary skills to care for small newborns and preterm babies. (Fed_12_PC).*

Most participants perceived staff have poor realisation responsibility, resulting in poor-quality health services or interruption in service delivery. Their supervisors cannot act on staff’s inaction. One local health officer said:

*Staff have poor realisation of their responsibility. Many health workers remain in the health facility from 11 am to 2 pm; staff come late but leave offices earlier. Staff are found to be talking about personal things while service users are queuing. Such an act might hamper delivery of essential health services, including childbirth services, in remote areas. (L_4_GO)*

The majority of study participants said providers’ language and cultural differences contribute to poor trust in public services and lead some women to seek care from traditional healers first, only using formal health services if the health issue continues.

*We cannot provide women-friendly services as we lack SBAs who can speak the local language and understand cultural values. (L_1_GO)*

Most study participants perceived health services often hold “victim-blaming” attitudes towards pregnant women, sometimes scolding them for not taking the advice of health staff (e.g., if they do not follow-up visits as recommended). One local officer said:

*If you asked health workers why pregnant women do not visit facilities, they would reply to service users who were not advised or had poor health awareness. Instead, women may not have visited facilities due to a lack of services (e.g., irregular and interrupted service). (L_1_GO)*

#### Poor empowerment in decision-making on health services (I)

Most study participants believed financial and sociocultural factors are contributing in poor access to health services. For instance, disadvantaged women (poor, rural areas, hills, and mountains) often have many household responsibilities and roles (e.g., family, productive and reproductive) due to male family members working as laborers overseas. These responsibilities can prevent women from prioritising accessing antenatal services. One NGO professional said:

*Women of remote areas do not get adequate support from family financially and emotionally during their pregnancy-postnatal period when male partners are abroad for employment. Back home, women lack access to and control of resources and decision-making and need to rely on the decisions of other family members (e.g., mothers-in-law) to reach the facilities. Such women cannot seek routine MNH services. (Fed_NGO_8)*

#### Inadequate community participation and engagement (S)

Most study participants felt local health committees often exclude disadvantaged communities (e.g., Dalits, Indigenous, or females) in the decision-making process, or engagement is tokenistic. One study participant explained that even where people from disadvantaged groups are included as committee members they may not be adequately listed to or heard:

*Some members of the local health committee (e.g., Dalit members) do not know their roles in those committees and cannot speak up for their communities. (Prov_6_NGO)*

To sum up, the local-level challenges were related to service delivery. Almost all study participants further explained challenges from the higher level.

### Provincial level challenges

Three multidomain challenges identified at the provincial level included weak decentralisation, lack of evidence uses and poor contextualisation of macro-health policies at the provincial level. Furthermore, most interview participants perceived inadequate resources and institutional arrangements to influence the challenges at the provincial level.

#### Weak decentralisation in the health sector (S)

Most study participants felt poor decentralisation of administrative and fiscal authorities are negatively affecting the delivery of health services. According to study participants, over two-thirds of the health budget is allocated to the federal level. One federal study participant said:

*Federal officers’ mindset is power centred and centralised, limiting the financial and administrative federation to lower levels. There is no clarity on the delegation of resource allocations and authorities to all levels of government. (Fed_3_MO)*

One reason given for lack of decentralisation in practice was a belief that local governments lack the capacity to enact their responsibilities. One health official said:

*The municipal governments need some technical support in health planning and management as these governments cannot execute the constitutionally mandated health rights. (Fed_1_GO)*

All study participants also felt that a “one-size-fits-all” was taken at the federal level, which did not consider local context and community needs. One local officer said:

*Current blanket policies cannot contextualise local issues to address the real problems. Federal-level key informants could not understand the local contexts (e.g., local issues and resources)*. *The top-down approach is like giving food to sparrows in a small funnel. Federally designed program strategies might be inappropriate and unacceptable for addressing local problems. (L_6_GO)*

#### Inadequate evidence-informed programs (HS)

According to study participants, local evidence and context on the causes of local problems and priorities are not taken into account, hampering the operationalisation of lower-level macro-health and addressing equity gaps. One study participant explained:

*Without local evidence, we cannot solve health needs and address the widening gaps in MNH services, which require data-driven contextualisation of program planning and implementation. There are practices of designing programs and adding some activities to last year’s budgets. (Fed_8_NGO)*

Some study participants discussed the lack of objective measures of marginalisation status of people (e.g., wealth status or income), that could inform health service delivery. Instead, they said, subjective measures (e.g., ethnicity, geography) of marginalisation were used, ignoring the fact that there could be wealthier and more privileged people in marginalised ethnic groups or remote areas. Therefore, the routine health management information system data was not seen as a credible indicator of service utilisation among the most disadvantaged groups. For example, one provincial health officer said:

*Nepal lacks data on income-based objective measures of households. The subjective measurement of marginalisation (e.g., ethnicities and gender) is important but might be insufficient to contextualise the health policies. We lack data on utilisation of the status of their service among disadvantaged groups. (Prov_2_NGO)*

#### Inadequate contextualisation of non-health sector programs (I)

All study participants perceived a lack of health literacy programs designed and implemented in local contexts, cultures, and languages. Contextual health literacy programs could raise awareness of the need and availability of health services. One NGO professional explained:

*Women do not know the types of services available to health facilities and lack knowledge of their health needs. This is not the women’s fault who could not visit health facilities. But I think there is a lack of implementation of health in all policies (information, education) to improve health literacy. (Prov_6_NGO)*

Additionally, most informants felt a lack of contextualisation of health interventions other than health sectors which could address social determinants of health. Using m-health interventions could improve health literacy and address complex health needs in the digital era. One study participant said:

*We need to offer alternative strategies to increase awareness and demand for health services in changing contexts. Alternative health education and communication strategies must be designed using digital technologies. (Fed_4_UN)*

### Challenges at the federal level

Overall, challenges at the provincial level are influenced by several macro-level challenges and hinder operationalisation and implementation of macro-policies. Following macro-level challenges are influencing policy and governance at all levels contributing to health inequity.

#### Inadequate collaboration for health in all policies (I)

All study participants felt remoteness and poor transportation systems affect the daily life of women. They believed implementing infrastructure development (e.g., road networks) could address the difficult geographical terrain and transportation systems. However, the planning and implementation of macro-infrastructural developments are not aligned with public health policies in Nepal. One federal participant said:

*Reaching the health facilities is difficult as settlements are far in hills and mountains. The poor road network and transportation systems further complicate geographical inaccessibility. Disadvantaged women who usually reside far from facilities cannot access care when they need routine health care. (Fed_11_GO)*

Additionally, health facilities lack basic infrastructure in rural areas (e.g., buildings, accommodation, and shops) where women and their caretakers to access health facilities for services.

#### Poor health planning and management (HS)

Few federal level participants said there is poor health planning and management (e.g., poor health logistics management, workforce, fund management), which influences programs and services at the point of care. They further said there is a lack of reliable data on logistics and procurement, long lead times, and frequent shortages of essential commodities. For example, one officer working at the federal level said:

*Logistic management in health is quite complex and does not happen timely. It has centralised at the federal level. Poor transportation systems compound the frequent shortage of essential medicines and equipment (e.g., misoprostol, iron tablets for MNH services) at peripheral facilities. (Fed_11_GO)*

Furthermore, at the federal level, troublesome paperwork, and unresponsive bureaucracy (in procurement) could take a longer lead time resulting availability of health logistics. Few participants realised that central bidding and local ordering guidelines had not been implemented.

Most participants perceived that human resource management (e.g., promotion, transfer, deployment) is problematic and lacks performance management of workforces in Nepal. For instance, senior officers are frequently transferred from the Management Division (responsible for logistic management). In addition, the Nepal Health Service Act (1999) has provisioned the upgrading of workforces based on the total number of years they served rather than considering the qualifications and skills. One federal officer said:

*Health workforce management lacks a measurable performance management system, including qualifications and competency. Job descriptions are vague and subjective and not linked with a career-development plan. The transfers happen from the federal level without any assessment of local needs and do not have records (Fed_9_PA)*

Few participants raised concerns about the need for more health workers to address emerging epi-demographic problems; one local officer explained:

*Over the last three decades, our population has increased from 20 to 30 million. Still, health workforces are not increased in the public health system and lack proper forecasting and long-term human resource planning. There is a shortage of skilled birth attendants in rural facilities and no provision of graduate midwives in hospitals. (L_5_GO)*

Few local participants believed that there are issues (e.g., delayed budget release, low absorption of budgets, and duplications) in budget planning and management affecting service delivery. One local officer explained:

*The health budget is centralised and takes long- processes for approval. Budget lines duplicate in three layers of government. We must complete budgets allocated by federal levels first and expense all allocated funds at the end of the fiscal year. We need to work in a hurry to expense budgets and achieve the targets that might undermine the quality of MNH care. (L_1_GO)*

#### Weak digital governance and institutionalisation of health policies (S)

According to participants, while having a digital record-keeping system, it was not used consistently, and not all staff had the necessary capabilities or willingness to adopt the digital system. One study participant said:

*Investment in the digital system helps to keep the institutional memory can increase system performance (workforce performance) and efficiency. However, aged personnel lack the importance of digital systems and operating skills. There is digital infrastructure, internet connection and supply of power. (Fed_3_UN)*

Other challenges in not having a fully operational and up-to-date digital health information system were seen to be not having access to real-time data (e.g., services, funds), to track services and reduce corruption and not having information for evidence-based decision-making (e.g., planning, management, and service).

Some study participants also noted a long lag between policy-making and policy implementation into action and limited attention to the processes of institutionalising policy. One NGO professional said:

*There is a lack of commitment to translating political decisions into policy formulation. Parliament endorsed the Safe Motherhood and Reproductive Health Rights Act five years ago, but the implementation of this act has not been initiated yet at the MOHP. (Fed_15_NGO)*

A few participants noted how policy development and implementation often relied on individual personalities in policy-making or implementation positions. An example was given whereby if someone with a proactive attitude is in a decision-making position, then many policies and initiatives are initiated. The lack of institutionalisation, however, means that the replacement may not make much progress if an individual is transferred to another department. One focal person of MNH section of the UN agency said:

*Due to a lack of proper institutional mechanisms, personality-driven initiatives might not reach the implementation level. Look, the Family Health Division’s previous director initiated many promising maternal health policies (integration of MNH care), but once he was transferred, the current director came. No policy initiatives were not moving forward. (Fed_3_UN)*

#### Politicisation of health workforce (S)

All informants felt Nepal’s health sector had political influence at federal level (e.g., politically motivated trade unions of providers). All participants believed Nepal’s political party affiliated multiple trade unions, with their competing interests, hinders health system performance, as and are not apolitical as trade unions usually work for political parties, making union leaders very influential in policy decisions. One provincial level officer said:

*Health managers should work for trade union leaders’ interests; if not, they might be transferred from the post. Political party-affiliated trade unions may protest and force the manager’s transfer if they do not follow union’s demand. Their mother political parties always protect staff affiliating with a political party-based trade union. (Prov_6_NGO)*

#### Unregulated private health service (S)

Most participants stated privatisation had increased the choice of health services; however, the noted private health services are poorly regulated. For example, one informant working at the federal level explained:

*High out-of-pocket health expenses in private health facilities have become Nepal’s greatest threat to public health. I worry that catastrophic health expenditure will multiply as the country with a recently implemented health insurance system with low enrollment (private health services are not covered). Private health services will be one of the main reasons for indebtedness. (Fed_9_PA)*

Most participants said over-prescribing drugs and tests (even in maternity services) is common in private facilities (e.g., high C-section delivery rate without indications). For example, one study revealed C-sections (CS)conducted in private hospitals in Kathmandu were about 80% delivered in 2014 (far higher than the WHO recommended 5–15% of pregnancies who needs CS). This medicalisation and commercialisation of routine health services while people visit private facilities for perceived better quality health services. Privatisation of health services can create further economic burden on disadvantaged groups, who often do not have public health insurance. One federal health officer shared:

*Private hospitals in the capital city have the highest C-section delivery rates with increased hospital stay and inpatient care costs. Routine maternity services are over-medicalised with the commercial interest of private hospitals, which can burden poor women while accessing routine health services. (Fed_9_PA)*

#### Corruption and limited accountability in health sector (S)

All participants agreed that corruption, due to a lack of effective health system governance and accountability, is the major systemic factor affecting the health system’s effectiveness.

*In Nepal*, financial c*orruption (e.g., procurement of health commodities) is a major concern. Authorities would take it in exchange for buying the goods from vendors and suppliers. You read many health officials were suspended in the procurement of misoprostol last year. (L_2_GO)*

Poor health system governance was reported to influence staffing evidenced by nepotism. One retired health officer said.

*It will be okay if you do or do not; no one cares about your performance and health system is not functioning as per rules. Individuals are part of the system, which does not work in isolation. (Fed_10_Adv)*

Most study participants at federal and provincial levels felt governmental health regulatory bodies such as the Nepal Medical Council are weak and unable to undertake their regulatory role effectively. One informant explained:

*The governmental health regulatory bodies (e.g., Nepal Medical Council) are weak or poorly functioning. These institutions have more work on paper, but less regulatory actions have existed but poorly working. (Fed_10_Adv)*

Most key informants believed that macro-level challenges are politically linked, and health system governance reform is needed.

## Discussion

This study explored the systemic challenges influencing policy and governance to create equitable health services at multiple levels. Challenges identified at federal-level included poor health system governance, and weak health planning and management. At the provincial level challenges identified were poor decentralisation of power and resources and lack of evidence-based planning. The local level challenges were social exclusion and poor-quality health services. Overall, these challenges were related to (i) federally controlled policy implementation structure; (ii) inadequate provincial institutional arrangements per the constitution of Nepal; and (iii) the local health systems lack institutional and policy capacity for implementation. The challenges are highly contextual, can operate at policy, operational and implementation levels, and interact with structural, intermediary challenges operating in multi-level health systems.

### Municipal health system for addressing challenges of service delivery

After federalisation, the district health system transformed into the municipal health system but faced challenges both from the supply and demand side for program implementation, service delivery and increasing utilisation by disadvantaged groups [34, 46]. Other studies have identified supply-side challenges, such as a shortage of essential medicines and supplies prior to the implementation of federalised health system in Nepal [31, 47]. Such challenges are still existed after implementation of the federalised health system. To address these constraints, capacity of local community health systems should be strengthened to prioritise health problems, mobilise local resources and upgrade local health facilities (e.g., health workforces, and health infrastructure) for quality of MNH services.

The current study also indicates the need for more locally hired health workers with local cultural and linguistic knowledge positively influencing the provision of quality MNH services. Such health workers can collaborate with community leaders to identify unmet health needs of marginalised groups and ensure culturally appropriate health services [36, 48, 49]. For example, local health workforce has potential to empower and engage in effective coordination with local communities and improve accountability if they are included in the health facility management committee [50]. Local recruitment of health care providers with necessary skills, competence and motivation in combination with digital technologies, can potentially improve the quality of health services [51, 52].

Our study revealed health service delivery was hampered by poor community participation, especially of disadvantaged groups in health care decision-making. Community participation is one of the fundamental principles of PHC [53]. Empowerment and engagement of service users in the local health committee further enrich the accountability and implement grievances handling interventions which inform the service delivery towards desirable health outcomes [42, 54, 55].The municipal governments have authorities to execute the formal rules and regulations, community coalitions, and supply and demand behaviour of individuals and providers [56]. For this, decentralisation of authorities and budgets is essential and local communities also need to be aware of their role in local health governance [57]. Additionally, some potential strategies in the local health system could include community groups such as health mothers’ group, and the inclusion of socially disadvantaged groups (such as ethnic minorities, and differently abled people) in the health facility management committee[9, 58].

### Provincial health system for operationalisation of macro-health policies

This study indicates the decentralisation of the health system in Nepal is weak and poses challenges to effective and efficient implementation of MNH policies. Operationalisation of macro-policies needs to be informed by the provincial context. Previous studies suggest that providing more power to the provinces can enable more contextualisation of health services, and strengthen provincial health system governance (e.g., health organogram, staffing) by allowing the provinces to govern the municipal health systems and bringing accountability closer to implementing agencies [59, 60]. The role of the federal health system is oversight and regulation while provincial health systems operationalise macro-policies as per provincial contexts [60]. For this, decentralization of resources, authorities, and provision of essential capacity at the provincial level is vital for effective implementation of federal health policies.

The study also suggests at the provincial level; there is a lack of health institutions and workforce to operationalise macro-policies. For example, under the Federal MOH, there are health divisions (e.g., division for strategic policy and planning), and departments (department of health services and its program implementation entities). In principle, the role of the Federal MOH is to formulate policies and govern overall health systems; however, the DoHS under the federal MoH is currently working as an implementation unit at the federal level. There are no such provincial health institutions to operationalise and contextualise macro-health policies [15]. Moving organisational structure and responsibilities of DoHS to provincial level under the provincial health ministry could potentially support in implementation of health policies in the provinces.

The current study also identified inadequate evidence-informed health planning and programs at the provincial level. Currently, provincial health systems lack specific targets for their populations within the province, as there are ethnic and linguistic diversities. For example, in Madhesh province, most people speak Maithali, or Bhojpuri as their native languages. Thus, Madhesh provincial health system should consider such diversities in designing health programs by adopting provincial health strategy [11, 61]. Additionally, evidence-based and contextualised strategies at the provincial level can also address the underlying SDoH for improved MNH outcomes [62].

### Federal health system for macro-policies and governing frameworks

Participants identified the macro-level challenges in reducing inequities in MNH as being corruption, poor regulation of the private health services, weak accountability, politicisation of the health workforce. These challenges influence the lower-levels, including management, planning, operationalisation of policies and implementation of programs.

While progress has been made in developing heath policies, the study also identified gaps in regulations and policy implementation. For instance, there is a lack of a macro-level regulatory framework for private health services (e.g., quality and standards of care, care costs). In Nepal, PHC services (including MNH services) are freely available in public facilities but not private health facilities. The NHIP is being implemented, it lacks a policy for purchasing health services from private facilities [63]. Purchasing private services through insurance schemes could improve access to private health services for people from disadvantaged backgrounds in urban areas. Furthermore, the mandatory enrolment of public and private providers in the NHIP, and strict implementation of private health sector guidelines (10% beds to be allocated to disadvantaged groups) could also improve access of MNH services [18]. Evidence also supports that efforts in accrediting private providers and expanding social health insurance programs increased health service coverage among underserved populations in LMICs [64].

Our study identified challenges in health workforce governance, including politicisation and trade unionism of health workers. For instance, participants perceived a strong political influence on the health workforce (such as transfer and promotion), including midlevel workers (maternity care providers). Additionally, health workforce development and deployment remain highly centralised and politicised despite the federalised health system. Such activities can contribute to maldistribution of the workforce and frequent transfers, negatively influencing health programs and services management at local health facilities [65]. Interoperable digital tools could help with health workforce governance, monitoring and implementing regulations, improving accountability and reducing corruption [66]. Effective use of digital systems can mask policy expansion by using evidence, and big data analysis, enhancing their effective implementation and making a strong state-society favouring transparency, communication, and collaboration [67].

Furthermore, collaboration with non-health sector can be macro-level strategic lever in designing public health policies and ensuring health in all policies approach. For instance, strategic policies of non-health sectors can contribute to women’s and children’s health. Infrastructure development initiatives (e.g., physical infrastructure, transportation systems, roads and bridges) guided by principles of equity and social justice can influence the public health by improving living and working conditions of the populations and communities [68, 69]. Studies revealed that investing in girls’ education, water, sanitation and hygiene, poverty reduction, nutrition and food security, and infrastructure development effectively improved maternal and child health in many LMICs [70, 71]. Collaboration between the health and non-health sectors is often seen as a common-sense route to improving population health [72].

Nonetheless, strategic collaboration in designing health in all policies at the macro-level is essential for resource allocations and creating an enabling environment for multisectoral actions in health. Formal mechanisms and informal networks (working groups at the federal level) support to ensure enabling institutional environments (i.e. resources, management systems, structures) and underpinning values (e.g., ideas) [73]. Therefore, non-health sector development approaches require political commitment and accountability.

### Policy and institutional reforms towards equitable health systems

To reduce current inequities in MNH in Nepal, radical health system reform is needed that addresses these factors to ensure good health workforce governance, accountability, and regulation of private health services. Multiple reform strategies can be designed and implemented in the context of decentralised health systems. Firstly, the federal health system needs to focus on macro-level policy issues, especially good health system governance adopting digital tools, health workforce, regulation of private health services and ensuring accountability, and reducing corruption. Secondly, the provincial health system needs an operational department and divisions under the provincial MoHP (e.g., moving the current DoHS structure to the provincial level). For this, institutional reforms at the federal level could be the strategic move for developing provincial health policies and programs. The media, public interest groups, non-governmental and civil society organisations, and community groups also have a role to play in advocating for the implementation of decentralised health system. Thirdly, improving the provision of MNH services requires higher-level political commitment, institutional mechanisms, and technology-aided regulatory systems. The decentralisation in planning and management at lower-level governments can potentially strengthen health systems for equitable access to MNH services [74]. According to the provision of the constitution of Nepal, the previous district health system structure (e.g., local health facilities, including health posts) was transformed into the municipal health system in 2016 in line with the federal system [7].

**Fig. 2:**
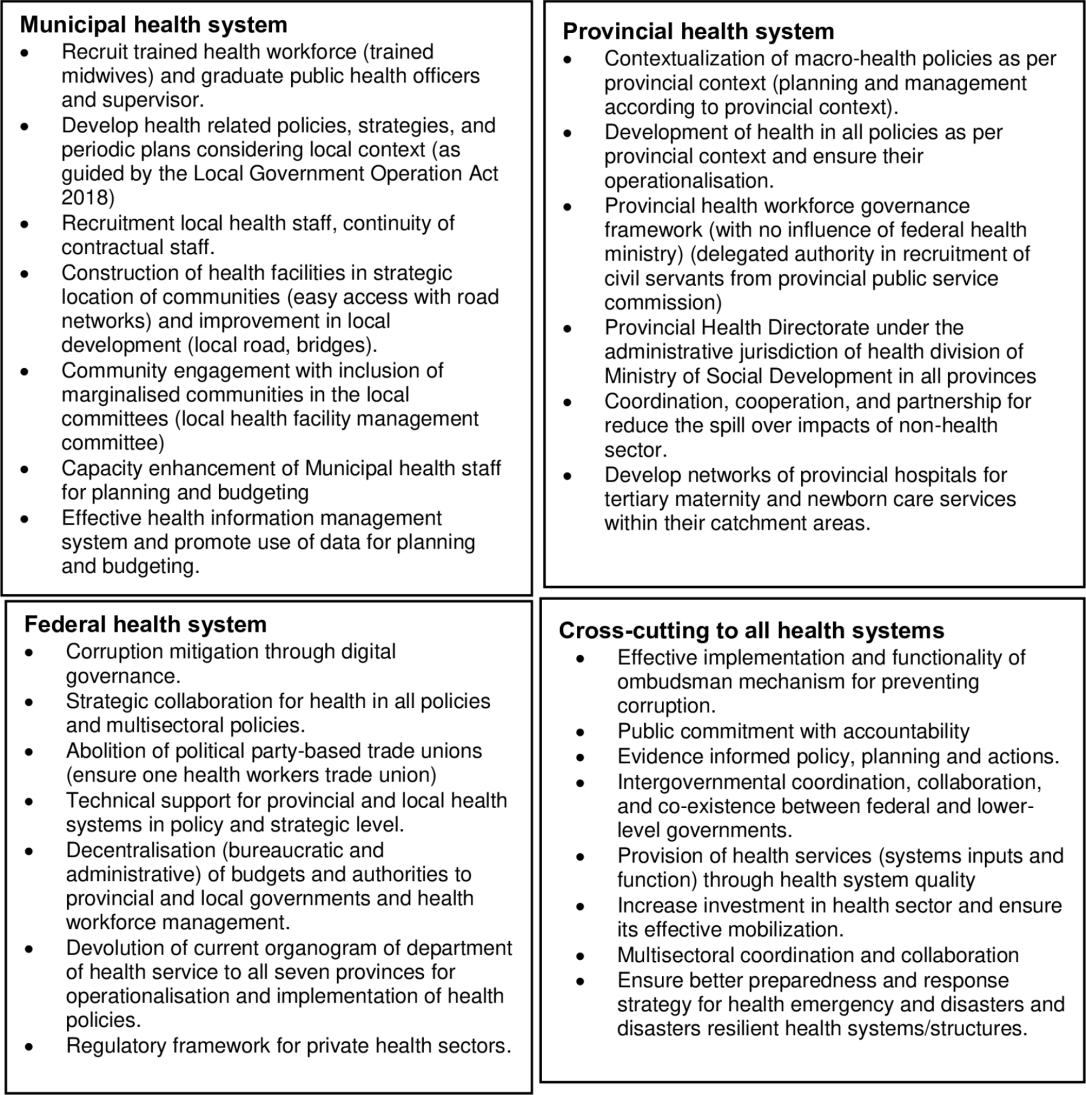
Potential health system reform strategies towards health equity in Nepal

Nevertheless, provincial and municipal health system has not been equipped and staffed was per mandate the constitutional mandates. Finally, local governments and governance can come together to debate local issues, including the contribution of non-health sector to health at the local level. Implementing decisions of local significance is an imperative component of a well-governed and responsive public sector and society [75]. Strengthening municipal health systems (capacity of local institutions and health workforces) is vital to addressing inequities in health and MNH services. Figure 2 presents some potential health systems reform strategies to improve health equity in Nepal.

This study has some strengths and limitations. First, study participants were from government, non-governmental organisations, international development agencies, academia, and professional associations with lived experience working at different health system levels. Secondly, this study provided a range of perspectives and challenges at multilevel health systems and how they operate at different levels. A limitation, however, is consumers were not included in the sample. Further qualitative research is suggested to understand local democracy and the adaption of policies and implementation challenges of the federal health system in different governments in Nepal.

## Conclusions

Over the last three decades, Nepal has made considerable progress in increasing access to health services, which has translated into reduced MNH outcomes. The PHC systems and the FCHVs have been pivotal to this success. However, equity gaps remain due to the complex interplay of micro, meso and macro-level underlying factors that intersect across multiple domains. Policy reforms are needed to influence these systemic factors at multiple levels to close the MNH equity gap and achieve the health SDGs. Such reforms need to delegate more power to the lower levels of the system and clarify roles and responsibilities at each level within the decentralised health system. Macro-level reforms and carefully designed supply-side interventions are needed to improve system performance at the local level. This will require political commitment with accountability. Better integration of accountability, transparency and anti-corruption mechanisms is needed to improve the health system governance [76]. The focus should be greater attention and resources to strengthen health systems to control corruption and promote transparency and accountability in the health sector [77].

The federal MOHP can provide policy guidance, while the provincial health system can contextualise those macro policies according to the provincial context, using evidence-based planning and implementation. Decentralisation depends on the context and associated complementary mechanisms and can enhance and exacerbate health-related equity. Features of decentralisation are central coordination, fiscal transfers and other equalisation schemes which have huge potential to enhance health-related equity [78]. In addition, decentralisation enhances equity with distribution of resources (voting with feet), bringing governance closer to people (close to the ground), and ensuring accountability (watching the watchers) [33].

At the local level, major reform is needed, including providing a supervisory team. The local health system needs a team of health workforce with program planning and management skills to support PHC. Multiple strategies can be implemented at the local such as improving health system responsiveness, enhancing community ownership, enabling the community to influence the policy decision-making process, the interaction between communities and the health system, and improving access to and use of MNH services [54, 79]. The municipal governments and facilities are closest to communities, they are well-placed to facilitate access to essential services and provide opportunities for citizen involvement in public affairs [75]. Provision of technical backup and management capacity needs to be ensured in each municipal health system. Provincial health institutions can function as operational levers for the local health system, while the federal health system can function as strategic levers to address inequity in MNH services in Nepal.

## Electronic supplementary material

Below is the link to the electronic supplementary material.

Supplementary Table S1: List of in-depth interview participants.
Supplementary Table S2: Descriptive characteristics of in-depth interview participants.
Supplementary Table S3: Interview guide for in-depth interview.

## Data Availability

Data used in this study is included in this manuscript.
