# Supplementary material for: Multidomain and multilevel strategies to improve equity in maternal and newborn health services in Nepal: perspectives of health managers and policymakers
Source: Int J Equity Health. 2023 May 26;22:105. doi: 10.1186/s12939-023-01905-7 (PMC10214695; doi:10.1186/s12939-023-01905-7)
Supplement: Supplementary file 1 — Supplementary Table S1: List of in-depth interview participants. Supplementary Table S2: Descriptive characteristics of in-depth interview participants. Supplementary Table S3: Interview guide for in-depth interview. [file 12939_2023_1905_MOESM1_ESM.doc]

Supplementary Table S1: List of in-depth interview participants

| **SN** | **Code** | **Organizations** | **Experience (years)** |
| --- | --- | --- | --- |
| 1 | Fed_GO_1 | Management division | 7 |
| 2 | Fed_GO_2 | Retired | 35 |
| 3 | Fed_UN_3 | UNICEF | 12 |
| 4 | Fed_UN_4 | WHO | 30 |
| 5 | Fed_GO_6 | Retired Chief. | 30 |
| 6 | Fed_BO_7 | DFID Nepal | 17 |
| 7 | Fed_NGO_8 | Save the Children | 17 |
| 8 | Fed_PA_9 | Kathmandu University | 35 |
| 9 | Fed_Adv_10 | Retired GO officer | 30 |
| 10 | Fed_GO_11 | Ministry of Health | 2.5 |
| 11 | Fed_PA_12 | Nursing Association of Nepal | 35 |
| 12 | Fed_NGO_13 | Helen Killer International | 25 |
| 13 | Fed_NGO_14 | Care Nepal | 10 |
| 14 | Fed_NGO_15 | One Heart Worldwide | 7 |
| 15 | Prov_NGO_5 | Plan Nepal | 30 |
| 16 | Prov_NGO_1 | Plan Nepal | 8 |
| 17 | Prov_GO_2 | Karnali Province | 6 |
| 18 | Prov_GO_3 | Karnali Province | 27 |
| 19 | Prov_NGO_4 | Karnali Province | 30 |
| 20 | Prov_GO_5 | Karnali Province | 19 |
| 21 | Prov_NGO_6 | Karnali Province | 23 |
| 22 | Prov_GO_7 | Far western province | 25 |
| 23 | L_GO_1 | Banke Sub metropolitan | 26 |
| 24 | L_GO_2 | DHO Jumla | 24 |
| 25 | L_GO_3 | Birendranagar Municipality | 20 |
| 26 | L_GO_4 | Rapti rural municipality | 12 |
| 27 | L_GO_6 | DHO Dailekh | 30 |
| 28 | L_GO_5 | DHO Banke | 9 |

Table S2: Descriptive characteristics of study participants included in the study, 2019.

| **Characteristics** | **Category** | **Numbers (N=28)** | **Percent** |
| --- | --- | --- | --- |
| Ethnicity | Brahmin | 14 | 50 |
|  | Janajatis | 4 | 14 |
|  | Chhetri | 10 | 36 |
| Organization | Government organization | 13 | 46 |
|  | Non-Governmental organisation | 7 | 25 |
|  | United Nations/ bilateral | 3 | 11 |
|  | Retired government officer | 5 | 18 |
| Level | Federal | 15 | 54 |
|  | Provincial | 7 | 25 |
|  | Local | 6 | 21 |
| Gender | Male | 24 | 86 |
|  | Female | 4 | 14 |

## Table S3: Key informant interview guide

| Background information | | | | |
| --- | --- | --- | --- | --- |
|  | Name:  Sex:  Position:  Experiences: years | Place of work: central/ provincial/local | Roles: Policymakers/managers/providers | Organization: GO/INGOs/NGOs/others |
| Questions | | | | |
| No | Questions | Possible responses (primarily health system) | Probing questions | Causes of the causes  (public policy and social determinants of health) |
| 1 | What are Nepal's current priorities, issues and problems of primary health care (focus on maternal and newborn health) services? | Supply and demand-side factors, e.g., socioeconomically disadvantaged group, remote areas, poor quality, equity | Why, how, and in what context are these issues- time, space and context? | Population- needs and expectation; governance (policy and non-health sectors); platforms (accessibility and organization of care); workforce (numbers, skills and support), tolls (equipment, medicines, and data) |
| 2 | Why are we unable to address inequity in health services in Nepal?  What are the main drivers of these inequities (and in what context, i.e., are the drivers different in different contexts/settings? | human resources issues (numbers and skills), supplies, transportation, geography, cultural practices or poor care-seeking, poor care-seeking, unavailability of services | Ask about underlying causes, why and how and in what ways these factors create inequities and responsible persons, | Lack of appropriate policies, Power centralization, and governance issues etc. Remember individual and institutional intersectionality, vertical (institutions) and horizontal (individual/family/community) equity. |
| 3 | What are the underlying factors that constraint equity in health services in Nepal | Blame game. Higher authority may doubt to local govt. | What happens if we do that, why do we need to do that action, etc.? | Socio-political level, developmental factors at a higher level |
| 4 | What solutions/ policies do you think would address those problems? How would it be possible to achieve equity in health services in Nepal? | Training, exposure budgets etc. | Probe asking how and why, and in what context in their response. | Contextual planning, decentralization, community engagement, use of digital technology |
